# Management and Prediction of Acute Pancreatitis Severity Using AI: A Surgical Perspective

**DOI:** 10.3390/diagnostics16091350

**Published:** 2026-04-29

**Authors:** Ioana Dumitrascu, Narcis Octavian Zarnescu, Giovanni Marchegiani, Alexandru Ilie, Eugenia Claudia Zarnescu, Radu Virgil Costea

**Affiliations:** 1 Department of General Surgery, “Carol Davila” University of Medicine and Pharmacy, 050474 Bucharest, Romania; ioana.dumitrascu@drd.umfcd.ro (I.D.); eugenia.zarnescu@umfcd.ro (E.C.Z.); radu.costea@umfcd.ro (R.V.C.); 2 Second Department of Surgery, University Emergency Hospital Bucharest, 050098 Bucharest, Romania; alexandru.ilie@rez.umfcd.ro; 3 Hepato Pancreato Biliary and Liver Transplant Surgery, Department of Surgery Oncology and Gastroenterology (DiSCOG), University of Padua, 35128 Padua, Italy; giovanni.marchegiani@unipd.it

**Keywords:** acute pancreatitis, artificial intelligence, severity prediction, machine learning, predictive models, surgery

## Abstract

Acute pancreatitis is a common inflammatory digestive disease with an unpredictable clinical course, ranging from self-limited forms to severe forms, associated with complications and increased mortality. Early identification of patients at risk of severe disease is particularly important from a surgical perspective, as it has a significant impact on subsequent management. Traditional severity scores, such as APACHE (Acute Physiology And Chronic Health Evaluation) II and BISAP (Bedside Index for Severity in Acute Pancreatitis), remain widely used, but their rigid structure and delayed applicability may limit initial risk assessment. In this review we highlight the evolving role of artificial intelligence in predicting the severity of acute pancreatitis and supporting clinical decision-making, with a focus on surgical management. Recent advances show that data-driven models could improve early risk assessment compared to traditional methods. Although their potential clinical benefits are becoming increasingly clear, real-world implementation remains limited. Initial results are encouraging, but important questions regarding reliability, safety, and integration into clinical practice still need to be addressed.

## 1. Introduction

Acute pancreatitis (AP) represents one of the most common inflammatory digestive diseases, with significant associated morbidity and mortality [1]. The global annual incidence rate of AP is 34 cases per 100,000 people [2]. Of these, approximately 20% represent severe cases of AP that can generate serious local and systemic complications, in-hospital morbidity and increased mortality, resulting in a mortality rate of 1.60 deaths per 100,000 people per year [2].

According to the 2012 revised Atlanta classification, AP is divided into three categories, depending on severity: mild (no complications or organ dysfunction), moderately severe (local complications or transient organ dysfunction), and severe (persistent organ dysfunction) [3]. Although most cases are mild or severely moderate and resolve in several days, the percentage of severe cases is the one that provides the most local and systemic complications, generating sustained organ dysfunction, sepsis, the need for interventional procedures, and increasing the length of hospitalization [4].

For surgeons, the ability to predict early which patients with AP will develop severe disease or necrosis can be an important aid. Such anticipation of the disease trajectory allows for timely intervention, optimizing outcomes and reducing the incidence of complications. Several validated scoring systems have traditionally been used to assess the severity of acute pancreatitis. The Ranson score is based on a set of 11 clinical and laboratory parameters collected on admission and within the first 48 h. The APACHE II (Acute Physiology And Chronic Health Evaluation) score takes a broader perspective, integrating physiological parameters and baseline health status to estimate disease severity. For a more rapid assessment at the bedside, the BISAP (Bedside Index for Severity in Acute Pancreatitis) score was developed using five variables available within the first 24 h (blood urea nitrogen, mental status, signs of systemic inflammation, age, and the presence of pleural effusion). The Glasgow-Imrie score assesses eight parameters within the first 48 h, while the Marshall score focuses primarily on the extent of organ dysfunction, particularly involving the respiratory, renal, and cardiovascular systems. These systems provide a classic framework for assessing acute pancreatitis, but their rigid thresholds, limited variables, and requirement for a time of up to 24–48 h often fail to capture the dynamic complexity of the disease [5]. This can translate into delayed recognition of patients at risk of severe complications, highlighting the need for more adaptive and accurate predictive tools.

Although the volume of studies exploring the applications of AI in acute pancreatitis is growing, most of them mainly emphasize the technical performance of predictive models, while the practical implications of these tools for clinical decision-making, especially from a surgical perspective, remain underexplored. Also, many studies report promising predictive accuracy, while providing limited discussion of important challenges, such as model interpretability, external validation, and the possibility of integrating these systems into clinical workflows, within the multidisciplinary management of acute pancreatitis [5,6,7].

Therefore, through this review, we aim to critically examine the current landscape of AI-based tools for the prediction of severity in acute pancreatitis, with a focus on their potential role in supporting surgical decision-making. The ultimate goal is to show how these emerging technologies can complement existing severity scoring systems and contribute to more individualized care of patients with acute pancreatitis.

## 2. Literature Search Strategy

**Figure 1:**
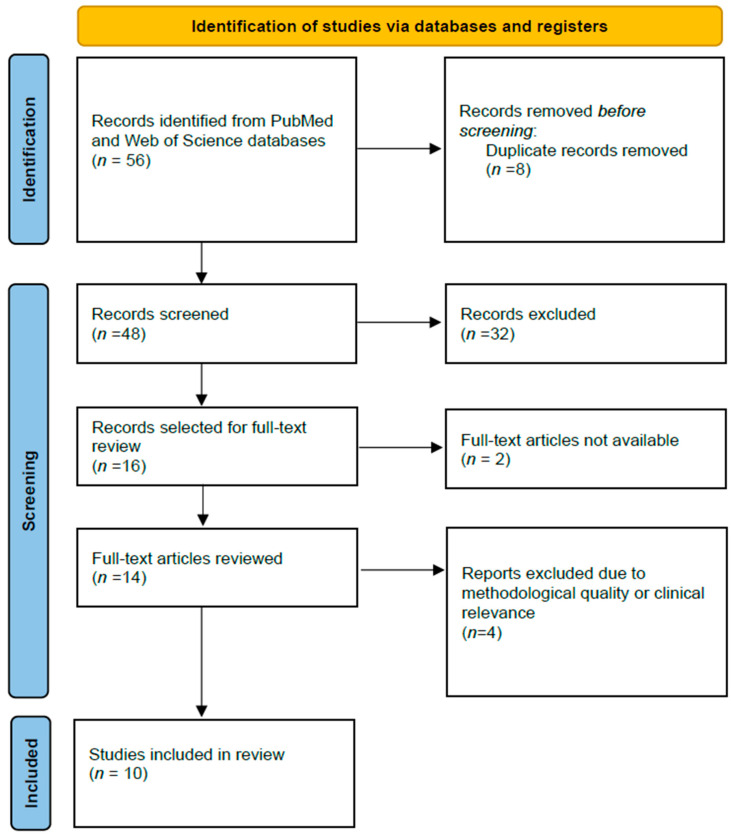
PRISMA flow diagram of the study selection process.

We performed a structured literature search using PubMed and Web of Science databases to identify studies evaluating the applications of artificial intelligence in acute pancreatitis, with a focus on severity prediction and clinical outcomes. The search strategy combined the following keywords: “acute pancreatitis” AND (“artificial intelligence” OR “machine learning” OR “deep learning”) AND (“severity” OR “prognosis”). All records retrieved from the databases were screened by title and abstract. A total of 56 records were identified by the database search. After removing duplicates (*n* = 8), 48 records were screened by title and abstract to exclude non-English publications, review articles, and studies not relevant to the applications of artificial intelligence in acute pancreatitis or its severity prediction. Of these, 32 records were excluded because they did not meet the inclusion criteria. Full-text articles were sought for the remaining 16 studies, but 2 could not be retrieved. Because this study was designed as a narrative review, a subset of 10 representative studies was ultimately selected for detailed analysis based on their methodological quality, clinical relevance, and use of advanced artificial intelligence models. The study selection process is illustrated by the PRISMA diagram in Figure 1.

## 3. The Role of the Surgical Team in the Management of Acute Pancreatitis

Surgery in acute pancreatitis is usually reserved for cases involving infected pancreatic necrosis, abdominal compartment syndrome, or complications that do not respond to conservative or minimally invasive surgical or endoscopic measures. Recent studies support the adoption of a step-up approach, which involves minimally invasive interventions staged before traditional open surgery [8,9,10]. A relevant example is the multicenter randomized PANTER (PAncreatitis Necrosectomy sTEp-up appRoach) study, which demonstrated significant advantages in mortality by using the step-up approach compared to classic necrosectomy in the management of pancreatic necrosis [11]. The timing of surgery is crucial: early surgery can exacerbate systemic inflammatory responses, while delaying surgery can lead to worsening sepsis or organ dysfunction [8]. In this delicate balance, AI can provide real support to surgeons by integrating large data sets from laboratories, clinical records, and imaging, thus contributing to more precisely identifying the appropriate time for intervention or personalizing treatment protocols [12,13].

## 4. Traditional Scoring Systems vs. AI Models in Severity Prediction

Traditional scoring systems, including the Ranson and APACHE II criteria, integrate clinical and laboratory parameters to stratify disease severity, with a sensitivity for predicting severity of 0.95 and 0.67, respectively, with an AUC of 0.95 and 0.94 [14]. Simplified bedside measurement tools, such as BISAP, provide rapid risk stratification but still require at least 24 h of admission, with a sensitivity of 0.67 [15].

However, from a surgical perspective, these tools have notable limitations because they rely on static variables and predetermined thresholds, often failing to reflect dynamic disease progression or discrepancies between clinical, biochemical, and imaging findings [5]. As a result, critical decisions regarding the necessity and timing of surgical interventions could be suboptimal. This gap highlights the need for more accurate, adaptive, and real-time predictive tools, such as artificial intelligence models, that can integrate multidimensional data to support individualized surgical decision-making [16]. Starting from this need, the field of artificial intelligence-based prediction involves continuous risk prediction, moving from classic scores to models that update as the patient progresses, allowing repeated recalculation of risk throughout the progression of the disease [7,16]. Table 1 shows a comparative overview of classical severity prediction scores vs. artificial intelligence-based predictive models used in acute pancreatitis.

For surgeons, this translates into earlier recognition of deterioration, better identification of patients who may benefit from minimally invasive or surgical interventions, and more confident decisions. Furthermore, explainable machine learning models have already been explored in the context of acute pancreatitis to predict mortality in the ICU, demonstrating how algorithmic risk assessments can complement clinical reasoning [17,18,19,20,21,22]. Sun et al. reported one of the most comprehensive prospective efforts to predict pancreatic necrosis infection in patients with acute necrotizing pancreatitis, developing an ensemble model that integrates a broad set of clinical and imaging variables. In their cohort of 1073 patients with acute necrotizing pancreatitis, the ensemble model achieved an AUC of 0.916 in the training cohort and 0.919 in the internal validation. Also, the predictive performance remained stable across disease stages (AUC 0.888 at <7 days, 0.906 at 7–14 days, and 0.901 at >14 days) and was confirmed in an external validation cohort (AUC 0.883) [18]. It is important to note that the model was developed using data derived primarily from a single tertiary center over a long inclusion period (2011–2023), while the external validation was performed in a relatively small independent cohort. In addition, the model incorporated a large number of predictors (31 variables identified through LASSO—Least Absolute Shrinkage And Selection Operator—regression), which may increase its complexity and potentially limit its ease of implementation in routine clinical practice.

## 5. AI Models for Severity Prediction in Acute Pancreatitis

Artificial intelligence is increasingly recognized as a potentially valuable tool in the management of acute pancreatitis, particularly for predicting disease severity and patient outcome. Artificial intelligence tools used in acute pancreatitis vary considerably in how they are constructed and how they can be applied in practice. Traditional machine learning approaches, such as random forests or gradient boosting, are based primarily on clinical and laboratory data and tend to be easier to interpret, especially when combined explainable techniques are used. In contrast, deep learning models are better suited for more complex data, such as imaging or continuously collected clinical information, but are often less transparent and more difficult to integrate into daily practice. These differences show that no artificial intelligence model is universally valid. These technologies have continued to evolve beyond conventional predictive models toward more advanced approaches that enable multimodal data integration, explainable clinical decision support, and continuous patient monitoring.

### 5.1. Data Types

The predictive capacity of artificial intelligence in acute pancreatitis depends on the quality and diversity of the data it receives. Among these, clinical and laboratory variables remain the backbone of the input data. Together with basic demographic data, they form the starting point for any model that attempts to estimate early risk. These are complemented by bedside observations of the patient available within hours of admission, such as the presence of SIRS (Systemic Inflammatory Response Syndrome) criteria, hemodynamic instability or persistent abdominal pain, which together provide essential context for the initial stratification [23]. Dynamic clinical measurements become more accurate as the disease progresses. Since patients with acute pancreatitis can deteriorate quickly and unpredictably, AI models use patient time outcomes in addition to static values to continuously update risk. The PANCREATIA study published by Villasante et al. [24] is an example of early AI-based risk stratification in acute pancreatitis. Using only clinical variables available at the time of presentation, without laboratory or imaging data, the authors developed AI models capable of predicting severe acute pancreatitis with good accuracy. Thus, in a prospective cohort of 594 patients analyzed, the authors developed two-stage models: the Stage 0 model, which, using clinical admission data, obtained an AUC of 0.698 for in-hospital mortality, 0.721 for ICU admission, and 0.707 for persistent organ failure. Adding vital signs to the Stage 1 model improved performance, with AUC values of 0.849 for mortality, 0.786 for ICU admission, and 0.783 for persistent organ failure, outperforming traditional scores such as APACHE-II and BISAP. In contrast, the moderate performance of the Stage 0 model indicates that clinical variables alone may not fully reflect the early biological complexity of acute pancreatitis. In addition, although the prospective design represents a methodological strength, further external validation will be required to confirm the generalizability of these findings.

One of the most crucial inputs for AI-based severity prediction is imaging data, particularly contrast-enhanced computed tomography, which captures anatomical and morphological features that cannot be deduced from clinical or laboratory variables alone. Pancreatic and peripancreatic inflammation, necrosis, fluid accumulations, vascular changes, and retroperitoneal tissue changes can be quantified by computed tomography and transformed into valuable input data. Qi et al. evaluated the role of early CT-based radiomics in predicting the severity of acute pancreatitis. Using patterns identified on initial CT scans, they developed machine learning models that achieved an AUC of 0.871 in the training cohort and 0.859 in the validation cohort. Combining radiomics features with clinical variables further improved performance (AUC 0.905–0.908), demonstrating that radiomics provides complementary information that improves early risk stratification [25]. Because the model was derived from a retrospective dataset of only 138 patients, the reported performance metrics should be interpreted with caution. Whether the model can generalize beyond this cohort remains to be established in larger populations.

Time-series electronic health record (EHR) data, represented by repeated, time-stamped measurements such as vital signs, laboratory results, fluid balance, medications and therapies, and timing of procedures, provide the raw material for dynamic, continuously updated risk models [26]. In studies dedicated to acute pancreatitis, modern artificial intelligence methods are integrated into multicenter infrastructures through an advanced distributed model optimization protocol, in which longitudinal clinical and paraclinical data streams are processed locally, and only model parameters are aggregated centrally. This decentralized design ensures the protection of sensitive data and improves the performance and transferability of early severity prediction. [27]. Therefore, time-series electronic health record (EHR) inputs enable continuous, individualized risk estimates that anticipate deterioration, prioritize early escalation, and more effectively predict the timing of surgical or minimally invasive interventions. Mesinovic et al. [16] introduced DySurv, a dynamic deep learning-based model that integrates static and longitudinal clinical data to continuously update individualized risk estimates. The model outperformed traditional ICU scores (APACHE and SOFA—Sequential Organ Failure Assessment) in predicting 24 h mortality, with an AUC(t) of 0.603, 12% higher accuracy and 22% higher sensitivity. Using time-dependent clinical information, DySurv represents a shift towards continuously updated risk assessment rather than a single point in time assessment. This perspective aligns with the dynamic clinical evolution often observed in acute pancreatitis.

### 5.2. Model Architectures

Random forests, gradient boosting machines (e.g., XGBoost), and support vector machines are examples of machine learning (ML) models that are effective at mixing structured clinical and laboratory data to find nonlinear associations that might not be apparent to physicians. These models might facilitate early triage by recognizing patients who may need intensive monitoring or early care [28]. They have shown significant predictive performance for outcomes such as organ failure, pancreatic and peri-pancreatic necrosis, and ICU admission. Li et al. [23] developed a machine learning model to predict 30-day mortality in acute pancreatitis using clinical and laboratory data. By applying SHAP (Shapley Additive Explanations), this study highlighted the factors (e.g., elevated CRP or creatinine) that determined the risk for each individual patient, making the predictions interpretable and thus illustrating how artificial intelligence can support early identification of high-risk patients. The model demonstrated good discriminative performance, with an AUC of 0.881. An important aspect of this study is the use of explainable artificial intelligence techniques, which help clarify how different clinical variables contribute to the model predictions and facilitate the interpretation of the results in a clinical context. The reliance on retrospective data may influence how well the model performs beyond the original study population.

Deep learning (DL) approaches, including convolutional neural networks (CNN) and recurrent neural networks (RNN), can process high-dimensional data, such as imaging and time-series clinical variables. CNN-based models applied to contrast-enhanced CT scans allow for the automatic extraction of radiomic features associated with peri-pancreatic necrosis or inflammation, while RNN and transformer-based architectures can model temporal trends of vital signs and laboratory tests. Recent studies have demonstrated the potential of deep learning to improve severity prediction in acute pancreatitis through automated analysis of imaging data. Zhang et al. [29] developed a CT-based model capable of detecting early pancreatic inflammation and necrosis, accurately predicting severe from mild cases and providing useful information for early risk stratification and surgical planning. In a large multicenter study, Cao et al. [30] developed PANDA, a deep learning model that uses non-contrast CT scans to detect pancreatic lesions, achieving high performance with AUC values between 0.986 and 0.996 for lesion detection in over 6200 patients and demonstrating a sensitivity of 92.9% and a specificity of 99.9% for lesion detection. Other approaches based on convolutional neural networks have extracted radiomic features from contrast-enhanced CT scans, capturing subtle tissue patterns that are difficult to quantify visually, and integrating these features with clinical variables to improve prognostic performance [31,32,33].

Dynamic or survival-based AI models use longitudinal EHR data to track how risk evolves over time, providing a more realistic picture of disease progression than scores based on a single point in time. Luo et al. used a GRU (Gated Recurrent Unit—a type of multi-task recurrent neural network designed to model sequences of data, such as vital sign trajectories or laboratory results) model to analyze irregular sequences of vital signs and laboratory results, thus enabling real-time prediction of impending organ failure [26]. The model was trained on over 1.9 million time-stamped observations from 13 645 patients and outperformed traditional machine learning approaches (e.g., random forest, XGBoost) in terms of performance. The utility of dynamic trajectory analysis of biomarkers was demonstrated by Ly et al. in a study of dynamic lipase models, who showed that high lipase variability and persistent elevations are strongly linked to higher mortality [34]. When combined, these techniques offer a time-sensitive risk framework that goes beyond individual evaluations, assisting physicians in recognizing decline and directing timely decisions for therapy escalation or surgical intervention. Despite their promise, these models face important limitations. They rely on large, high-quality longitudinal datasets and can be affected by missing or irregularly sampled data. External validation across diverse patient populations is essential before they can be widely adopted.

Multimodal and explainable AI frameworks integrate diverse streams of patient information, ranging from lab tests and vital signs to imaging findings and procedural history, into a unified model that reflects the full complexity of the patient’s condition. Rather than considering each data type in isolation, these systems analyze how different signals interact over time, capturing patterns that are often invisible to conventional scoring systems. Explainable AI methods, such as SHAP, reveal which features drive the model predictions, allowing clinicians to understand why a patient is flagged as high-risk and providing actionable insights for intervention [23,33]. Chen et al. [33] developed a model for predicting the prognosis of acute pancreatitis by combining CT-based radiomics with clinical data, illustrating a multimodal approach based on artificial intelligence. The combined model outperformed models based on imaging or clinical data alone, achieving AUC values of 0.899 in training and 0.877 in validation, thus providing more interpretable predictions. As shown in Table 2, AI models have shown promising results in severity prediction.

## 6. Applications of AI During the Management of Acute Pancreatitis

### 6.1. In the Emergency Department

In the emergency department, the main challenge in acute pancreatitis is the early identification of patients at risk of severe disease, especially when they are at a stage when symptoms may still be mild and nonspecific. Conventional scores, such as Ranson, BISAP, and APACHE II, are based on variables collected over 24–48 h, have moderate predictive accuracy, and a static approach to risk assessment. Therefore, artificial intelligence-based models have focused on early risk prediction at the time of admission. These models are based on a small set of variables, such as age, heart rate, systolic blood pressure, blood urea nitrogen, hematocrit, creatinine, C-reactive protein, and oxygen saturation, making them suitable for application in the emergency department [37]. For example, Thapa et al. [38] developed machine learning models based on early clinical and laboratory variables, achieving AUC values close to 0.90 for predicting severe acute pancreatitis and outperforming conventional scoring systems such as BISAP and APACHE II. More recently, Chang et al. [39] demonstrated the feasibility of implementing real-time artificial intelligence in the emergency department, reporting AUC values exceeding 0.95 for ICU admission and mortality prediction, with clear superiority over BISAP. It is important to note that these models achieved high discriminatory performance without relying on imaging or advanced biomarkers, supporting their applicability in emergency settings.

### 6.2. Inpatient Ward

After admission to the ward, the crucial step is not diagnosis but anticipating which patients will deteriorate, as even those initially classified as mild or moderately severe may develop persistent organ failure, pancreatic necrosis, or systemic complications within the first 48–72 h. AI models trained on longitudinal clinical data (e.g., vital signs, laboratory trends, fluid balance) can continuously update risk predictions and detect high-risk trajectories before overt clinical deterioration occurs. As previously discussed, Luo et al. [26] applied a time-dependent recurrent unit (TRU) model to Electronic Medical Record (EMR) data of hospitalized patients, demonstrating that dynamic AI can predict organ failure and progression to severe disease with high accuracy (AUC 0.88–0.92), outperforming repeated application of APACHE II or BISAP. A recent meta-analysis by Yuan et al. [40] showed that AI-based early warning systems significantly improve patient outcomes, including reduced in-hospital and 30-day mortality, by alerting physicians to clinical deterioration earlier than traditional methods. Importantly, the analysis included studies in which the prediction models had already been evaluated through prospective clinical validation. The included studies varied in terms of patient populations, outcome definitions, and model characteristics, which should be considered when interpreting the pooled results. In addition, most studies were conducted in single-center or nonrandomized settings, which makes it difficult to determine how consistently these systems would perform when implemented across different clinical workflows.

### 6.3. CT-Guided Management

In acute pancreatitis, contrast-enhanced computed tomography remains the cornerstone for assessing disease severity and is performed in several stages: at the baseline, for monitoring during hospitalization or for follow-up. Artificial intelligence-based tools can improve each stage by providing quantitative analyses that complement the radiologist’s interpretation. At the initial CT scan, convolutional neural networks and radiomic models can map the pancreas and surrounding fluid collections, generating detailed quantitative information about tissue structure and inflammation, which can support early prediction of severity, with Pan et al. reporting an AUC above 0.90 [35]. During subsequent CT scans, artificial intelligence can track changes in necrosis and fluid collections, integrating imaging and clinical data to support prognosis and detect patients at risk for late complications [33].

### 6.4. Integration of AI into Surgical Decision

The integration of artificial intelligence into surgical practice promises to profoundly reshape the way clinical decisions are made through its ability to analyze large volumes of data, identify subtle patterns, and provide rapid estimates of risks and outcomes. In severe acute pancreatitis, determining when to switch from conservative to invasive treatment remains a difficult surgical decision. Current guidelines support a step-up approach, with intervention being delayed whenever possible until necrotic collections are well delineated. In practice, however, this decision may be difficult in patients with persistent organ failure or suspected infected necrosis. In such situations, AI-based predictive models can help refine risk assessment. For example, models that predict infected pancreatic necrosis can help identify patients who are more likely to require drainage procedures [18], while algorithms developed to predict severe disease or short-term mortality can highlight patients at higher risk of clinical deterioration [23,38]. An important aspect is that their use requires careful interpretation. Overestimation of disease severity could lead to premature intervention, and underestimation of risk, in contrast, may delay necessary treatment. Therefore, AI tools should be considered decision support systems that complement the clinician/surgeon’s judgment [41].

Although AI models are frequently developed and validated in experimental settings, only a small fraction reaches real-world clinical implementation because many are not initially designed to be integrated into real-world workflows [42,43]. Recent methodological guidelines, such as TRIPOD-AI and DECIDE-AI, emphasize the need to design studies with explicit implementation intent, including requirements for external validation, transparency, and integration into clinical workflows to overcome the barrier between algorithmic performance and real-world utility at the patient bedside [43,44,45]. A notable example of successful implementation is that of an AI model developed for the prediction of one-year mortality in patients undergoing colorectal surgery, which was not only prospectively validated but also integrated into daily clinical practice, supporting surgeons in risk stratification and tailoring therapeutic decisions in a personalized manner [46]. This type of integration demonstrates the potential of AI to tangibly support surgical decision-making when developed and implemented according to robust standards.

A new concept in AI applications in surgery is Surgomics, which involves combining multiple types of data (e.g., genetic, imaging, histopathological and intraoperative) to improve patient assessment and therapeutic decisions. Mtoor et al. [47] recently published a comprehensive review, proposing an “AI-enabled Surgomics” framework, based on the integration of genomics, radiomics and pathomics into common predictive models. The authors highlight that multimodal use of data can increase the accuracy of predictions but emphasize the need for methodological standardization and external validation. They also describe the concept of AiRGOS (Artificial Intelligence Radiomics, Genomics, Oncopathomics and Surgomics) [48], a model for stepwise integration of data into a uniform AI architecture. AiRGOS proposes a structure in which information from imaging, molecular profiling, digital histopathological and surgical data are combined into a single analytical flow, to support risk prediction, disease characterization and decision support. It is important to emphasize that AiRGOS currently represents a conceptual and development framework, not an implemented system, but it provides a direction for future multimodal AI models. Even though this type of concept is promising, its direct applicability to acute pancreatitis remains limited, as it requires rapid decisions and time-consuming data acquisition, such as genomic or histopathological analysis, which is often not feasible in the acute setting.

An example of an application already available is Cholecystectomy AI for iOS. The application uses a machine learning AI model to analyze intraoperative images in real time during laparoscopic cholecystectomy, estimating whether critical structures have been correctly identified https://apps.apple.com/lv/app/cholecystectomy-ai/id1519271391 (accessed on 26 April 2026).

Notably, only a limited number of studies have focused on assessment criteria directly relevant to surgical decision-making in acute pancreatitis. These include the prediction of infected pancreatic necrosis [18], a key indication for invasive intervention, as well as models that estimate the likelihood of requiring procedures such as percutaneous catheter drainage or necrosectomy [21]. Although still relatively few, these studies suggest that artificial intelligence could go beyond general risk stratification to more intervention-oriented decision support. Currently, this remains an important gap in the literature and a key direction for future research.

We argue that when properly integrated into clinical practice, AI systems can complement, but not replace, surgeon judgment in the management of acute pancreatitis. They can provide objective estimates of the risk of severe complications, mortality, or the need for surgery, and can suggest alternative therapeutic strategies. Several reviews show that AI should support surgeon decision-making through predictions and transparent data, without substituting medical expertise [49,50,51]. In practice, the potential of AI is recognized, but skepticism and limited familiarity with it slow down its adoption. The main obstacles include a lack of trust, difficult integration into clinical workflows, and the perception that AI does not fully reflect the complexity of the course of acute pancreatitis [51]. Widespread adoption requires education, increased confidence, and clear evidence of patient benefits [52].

### 6.5. In ICU

In the ICU, AI-based models are designed to handle multimodal, high-dimensional data streams that reflect the dynamic evolution of acute pancreatitis. These models integrate continuously monitored physiological, serial laboratory measurements and treatment-related variables, including vasopressor use, fluid balance, and renal replacement therapy. Using boosting architectures and deep neural networks, AI models have demonstrated superior performance over conventional scores in predicting critical outcomes such as persistent organ failure, sepsis, and ICU or 30-day mortality in critically ill pancreatitis patients [23].

Several studies focused on the management of acute pancreatitis in the ICU illustrate this stage-specific utility. For example, Jiang et al. [17] developed a gradient boosting model using ICU admission data to predict ICU mortality in patients with acute pancreatitis, achieving superior discrimination compared with APACHE II. The model was developed and internally validated within a cohort composed exclusively of critically ill ICU patients, allowing for a focused evaluation of machine learning performance in this high-risk subgroup. Similarly, Wei et al. [36] reported that machine learning models incorporating early ICU physiological and laboratory data accurately predicted in-hospital mortality, supporting risk stratification shortly after ICU admission. These models are particularly relevant in the initial phase of ICU care, where early identification of patients at high risk of rapid organ damage can help escalation of monitoring intensity, earlier initiation of vasopressor therapy, or proactive organ support [53].

In the development of severe acute pancreatitis in the ICU, septic complications represent a critical turning point in the course of the disease. In many patients, sepsis develops secondary to infection of pancreatic or peripancreatic necrosis and frequently requires interventional control of the source. A recent review [54] highlights the marked biological and clinical heterogeneity of sepsis, which continues to be a major factor in organ dysfunction and mortality in critically ill patients. This complexity has sparked interest in AI-based approaches for early detection of septic deterioration in ICU patients. In this context, a recent multicenter retrospective cohort study, led by Tranchellini et al. [55], evaluated deep learning-based sepsis prediction models in three large ICU datasets (HiRID, MIMIC-IV, and eICU), comprising over 200,000 ICU admissions. The authors showed that models trained on a single dataset often lose accuracy when applied to external cohorts, a phenomenon known as distribution shifting, which reflects differences in patient populations, clinical practice, and data recording across institutions. Retraining the models using local institutional data substantially improved predictive performance. Other recent studies using large datasets from the ICU have also shown that machine learning models that integrate longitudinal physiological and laboratory data can detect early patterns that precede clinical sepsis [56,57]. In severe acute pancreatitis, such approaches may facilitate earlier recognition of infected necrosis and guide source control interventions.

Beyond early risk stratification, AI models can also support ongoing ICU management by allowing for repeated risk assessment. Dynamic models incorporating longitudinal data are able to capture subtle changes in vital signs or laboratory values that may precede overt clinical deterioration. Studies using time-series models and gradient boosting approaches have shown that capturing these evolving patterns can help clinicians anticipate critical events, monitor patient trajectories, and adjust interventions in real time, highlighting the value of trajectory-based modeling in guiding care for critically ill patients [58,59,60].

### 6.6. In Follow-Up

**Figure 2:**
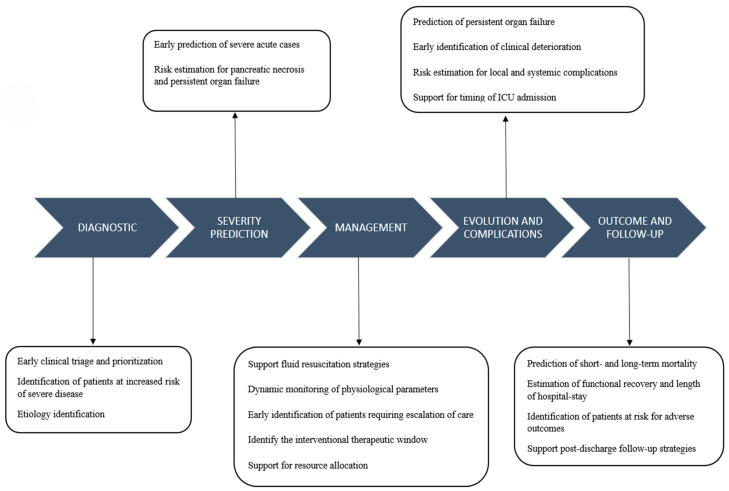
AI support during the course of acute pancreatitis.

Beyond the acute phase, AI-based tools can support structured follow-up and institutional learning in acute pancreatitis by analyzing outcomes in patient cohorts [61]. Post-discharge data can be used to identify factors associated with long-term adverse outcomes. For example, multi-cohort machine learning studies have demonstrated that inpatient data can be used to predict mortality up to one year after discharge [62]. Also, applied retrospectively, AI models can assess the impact of clinical decisions made during hospitalization, such as the timing of transfer to the ICU, initiation of invasive interventions, or surgical management. The ongoing MINERVA project [63] in Italy aims to develop and validate a machine learning model to predict the risk of recurrent acute biliary pancreatitis at 30 days, 60 days, 90 days and up to one year after admission. The project combines retrospectively and prospectively collected clinical and demographic data from multiple hospitals, with the goal of identifying patients at increased risk of recurrence (https://minervaproject.org/). Figure 2 illustrates how AI can provide support at each stage of acute pancreatitis management.

## 7. Safety and Limitations of Using AI in Surgical Decision-Making

In acute pancreatitis, the surgical decision is conditioned by the correct assessment of the severity of the disease, the identification of the progression to infected pancreatic necrosis and the occurrence of persistent organ dysfunctions, essential elements for risk stratification. Faced with the limitations of traditional clinical scores and the variability of imaging data interpretation, artificial intelligence represents an important decision support tool.

The decision to perform surgery in acute pancreatitis requires careful consideration due to the critical importance of timing and the potentially severe consequences of errors. Early intervention can prevent complications, but may also worsen systemic injury, while delayed surgery risks progression to infected necrosis or sepsis [64]. Decisions are also based on the anticipation of disease progression, and this progression may be uncertain [38]. Poor planning can increase the risk of morbidity and mortality, therefore careful and accurate prognostic assessment is important [65]. In this context, the integration of AI as a decision-making support tool must prioritize safety because any error could exacerbate the already high clinical condition [66].

In the management of acute pancreatitis, the contribution of artificial intelligence is not only represented by a “yes or no surgery” indication, but by specific decision support for delicate aspects and critical moments of the surgical part. Also, the safety of using artificial intelligence cannot be reduced to achieve high statistical performances. In practice, an AI model is only safe when its predictions remain reliable, explainable, and clinically relevant [67].

The first essential element of certainty is the robustness of the prediction. Youssef et al. emphasize that external validation of AI models is important but insufficient to guarantee clinical safety, so AI systems should undergo recurrent local validation to ensure that predictions remain robust, reliable, and clinically relevant in the specific population and medical context in which they are implemented [68]. Models developed on limited or homogeneous cohorts may produce good results in the internal validation phase but may fail when applied to patients with different clinical profiles [69]. Another important element is reproducibility, so that the predictions are consistent and stable over time. Interpretability is also particularly important. In the context of acute pancreatitis, surgical decisions can have major consequences, and the surgeon must be able to understand why an artificial intelligence model indicates a certain level of risk. “Black box” models can generate statistically correct predictions, but from a clinical perspective, they can be problematic because the underlying decision-making process is opaque, and the surgeon cannot see what features or patterns the algorithm uses to arrive at its recommendation [70]. As Amann et al. show, explainability in AI models is essential in medicine because it allows doctors to understand the rationale behind algorithmic predictions, critically evaluate AI recommendations, and safely integrate them into the management of each patient [71].

Safety in AI also means avoiding bias. Bias occurs when an AI system consistently overestimates or underestimates the risk for certain groups of patients. Ratwani et al. argue that continuously monitoring and correcting bias is particularly important to ensure that AI tools provide reliable and safe information [72]. Automation bias is a real safety concern in surgical decision-making for acute pancreatitis. Khera et al. raise the alarm that physicians may over-rely on AI-based decision support and have limited critical appraisal of the suggestions coming from AI models, especially in high-pressure situations [73]. In acute pancreatitis, this may lead to inappropriate escalation of the step-up approach or delay of intervention. Given the narrow therapeutic window in acute pancreatitis and its potential for serious complications, human supervision and the physician’s final decision remain essential to ensure the safe use of AI. Also, it is important to note that surgical readers should be cautious when interpreting AUC values approaching 1.0, as model performance frequently declines when applied to external cohorts, again illustrating the importance of external validation.

We conclude that perhaps the most important principle of safety is to preserve human decision-making control. AI should support surgical or medical reasoning, not replace it. The final decision should remain the responsibility of the physician, who integrates the information conveyed by the AI model into the clinical context of the patient.

## 8. Ethical Considerations

The use of AI in surgical decision-making, including in the management of acute pancreatitis, raises specific ethical considerations that go beyond algorithmic performance. Adherence to the fundamental ethical principles of autonomy, beneficence, non-maleficence, and justice requires that AI systems enhance, not replace, surgeons’ judgment, ensuring that critical decisions, such as the timing of intervention, remain under human control. Zhang et al. point out that bias, lack of transparency, and unclear accountability can make medical AI less trustworthy and can affect both surgeon control and patient experience [74]. It is important that AI-based decisions are easy to understand to ensure that patients can give informed consent and maintain a strong clinician-patient relationship [75,76]. Data governance is also equally important. The collection, storage, and use of clinical and preclinical data must comply with privacy regulations and ethical standards. Transparent policies and accountable protocols ensure that patient data are protected and that algorithmic outputs can be audited, supporting both safety and ethical integrity [71,77,78].

## 9. Conclusions

Acute pancreatitis can have an unpredictable course and early identification of patients at risk of severe disease remains essential from a surgical perspective. Standard scoring systems can provide guidance, but are often limited by their rigidity and delayed applicability.

AI models promise to improve early risk assessment and support surgical decision-making by analyzing complex patient data over time, complementing rather than replacing clinician judgment. Despite encouraging results, AI is not yet widely implemented in clinical practice. Key challenges include ensuring model reliability, interpretability, and secure integration into clinical workflows. Ethical considerations such as transparency, accountability, and patient autonomy must remain central when introducing AI into care.

Future research should focus on prospective clinical validation, developing user-friendly interfaces for medical-surgical teams and studying how AI can actively guide the timing and choice of interventions. Multidisciplinary collaborative efforts will be essential to ensure that AI tools are not only technically robust but also practical, safe, and meaningful in improving outcomes for patients with acute pancreatitis.

## Figures and Tables

**Table 1:** Traditional scoring system vs. AI models.

Characteristics	Traditional Scoring Systems	AI-Based Models
**TYPE OF DATA USED**	Limited clinical and biochemical data	Complex multimodal data
**AUC**	Moderate, global prognostic oriented ~0.78–0.85 [11]	Superior, can incorporate multiple and dynamic variables ~0.97 [12]
**ADAPTABILITY**	Fixed criteria	Dynamic
**USE OF IMAGING DATA**	Limited—CTSI	Extensive—radiomics
**REAL-TIME PROCESSING**	No	Yes
**PREDICTING THE NEED FOR SURGERY**	Limited, based on clinical course and standard imaging	Can predict the risk of local complications
**PREDICTION OF THE OPTIMAL TIME FOR INTERVENTION**	Empirical, guided by repeated clinical reassessments	Real-time prediction, with estimation of optimal interval for drainage or necrosectomies
**IDENTIFYING PATIENTS AT RISK FOR INFECTED NECROSIS**	Based on clinical signs/CT, with low sensitivity in the first few days	Early detection of infected necrosis through AI, analyzing subtle patterns in the laboratory and imaging
**COMPLEXITY**	Low, use fixed scores	High, requires computing infrastructure and multimodal data integration
**INTERPRETABILITY**	Easy interpretable	Variable—some models require explanatory tools
**IMPACT ON THE DECISION OF MIS VS. OPN**	Depends predominantly on physician expertise	May suggest optimal approach (e.g., endoscopic vs. percutaneous vs. surgical)
**LIMITATIONS**	Limited data integration Poor accuracy	Needs extensive data Risk of bias Regulatory hurdles

**Table 2:** Review of studies applying AI in acute pancreatitis severity prediction.

First Author, Year	Design	N	AI Tool	Target Outcome	Input	Performance (AUC—Best Reported Model)	Validation	Clinical/Surgical Relevance
Villasante S [24], 2024	Prospective	594	Ensemble ML (GB, RF, XGBoost)	Very early prediction for SAP, ICU admission, and mortality	Clinical data	SAP: 0.783	Internal	Predicts SAP, ICU admission, and mortality for early triage and management
Pan X [35], 2024	Retrospective	1124	CNN (MSAnet)	Pancreas segmentation and CT-based AP detection	CT images	0.990	Internal	Assists in rapid and accurate AP diagnosis
Qi M [25], 2024	Retrospective	246	Radiomics-based ML (SVM, Random Forest, logistic regression)	Early AP severity prediction	CT radiomics + clinical data	0.905	Internal + External	Predicts early severe AP for clinical guidance
Zhang C [29], 2024	Retrospective	437	AutoML (gradient boosting)	AKI prediction in AP	Clinical + laboratory data	0.993	External	Predicts AKI in AP to support early intervention
Li X [23], 2024	Retrospective	499	Explainable ML (XGBoost + SHAP)	30-day mortality prediction	Clinical + laboratory data	0.881	Internal cross-validation	Predicts 30-day mortality for clinical decision-making
Chen H [33], 2025	Retrospective	344	Combined radiomics–clinical machine learning	AP prognosis prediction	CT radiomics + clinical data	0.899	Internal + External	Predicts AP outcome to optimize management
Jiang M [17], 2025	Retrospective	1782	Explainable ML (XGBoost + SHAP)	ICU mortality prediction	Clinical + laboratory data	0.89	Internal	Predicts ICU mortality to support ICU management
Wei S [36], 2025	Retrospective	1802	Ensemble ML + explainability (XGBoost, RF, LightGBM + SHAP)	In-hospital mortality prediction	Clinical + laboratory data	0.835	Internal + External	Predicts in-hospital mortality in ICU patients
Li Y [34], 2025	Retrospective	834	Time-series ML	Dynamic mortality prediction (lipase-based)	Dynamic Lipase Trajectories	NA	Internal	Predicts dynamic mortality risk for early intervention
Cao J [20], 2025	Retrospective	732	LNN	Early SAP prediction	Clinical + Temporal data	0.965	Internal + External	Predicts early severe AP to guide treatment

N = number of patients; SAP = severe acute pancreatitis; AKI = acute kidney injury; ICU = intensive care unit; ML = machine learning; GB = gradient boosting; XGBoost = eXtreme gradient boosting; RF =random forest; CNN = convolutional neural network; GBM = gradient boosting machine; LNN = liquid neural network; AP = acute pancreatitis; AUC = area under the curve.

## Data Availability

No new data were created or analyzed in this study. Data sharing is not applicable to this article.

## Abbreviations

The following abbreviations are used in this manuscript:

AI	Artificial intelligence
AP	Acute pancreatitis
